# The Utility of Grip Strength as a Simplified Measure of Frailty in the Older Adult in the Preoperative Clinic

**DOI:** 10.7759/cureus.28747

**Published:** 2022-09-03

**Authors:** Dominique Spiegowski, Lia Metzger, Ankita Jain, Mario A Inchiosa, Garret Weber, Apolonia E Abramowicz

**Affiliations:** 1 Anesthesiology, New York Medical College, Valhalla, USA; 2 Anesthesiology, Beth Israel Deaconess Medical Center, Harvard Medical School, Boston, USA; 3 Pharmacology, New York Medical College, Valhalla, USA; 4 Anesthesiology, Westchester Medical Center, Valhalla, USA

**Keywords:** pre-operative evaluation, hand grip strength, cfs, frail, clinical frailty

## Abstract

Objective

The aim of this study was to compare the measure of grip strength against other validated methods of measuring frailty.

Materials and methods

This was a single-center, cross-sectional study that took place at the Westchester Medical Center Pre-Procedural Testing Clinic. The patient population included n = 73 patients ≥65 years of age evaluated for elective surgery. During the study, patients’ grip strength, CFS-I (Clinical Frailty Score of Investigator), CFS-P (Clinical Frailty Score of Participant), and FRAIL (Fatigue, Resistance, Aerobic capacity, Illnesses, and Loss of weight) scores were measured.

Results

Grip strength correlated negatively with the CFS-I, CFS-P, and FRAIL scores for females. Reduced grip strength in females correlated with higher frailty scores and vice versa. Male grip strength showed no significant relationship with the frailty scales. In addition, multivariate linear regression analysis revealed that the independent measure that demonstrated a significant inverse association with grip strength was age (β= -0.43, p = <0.001).

Conclusions

There exists a difference in the utility of grip strength as a measure of frailty between males and females.

## Introduction

Frailty is defined as a clinical state of vulnerability to endogenous and exogenous stressors that produces exacerbated outcomes when compared to non-frail counterparts [1]. It has been correlated with increased morbidity and mortality with exposure to stressors such as surgery [2]. Pre-operative frailty assessments in elderly patients have been used to predict adverse surgical outcomes, including prolonged hospital stay, all-cause mortality, morbidity, and discharge into skilled facilities instead of home [2,3]. Furthermore, elderly patients with elevated frailty are more likely to experience death or disability after surgery [4]. Thus, finding and implementing objective, comprehensive measures of frailty preoperatively is critical for risk stratification. Although there exist validated assessments of frailty with predictive value regarding postoperative outcomes, including the Fried Phenotype, Edmonton Frail Scale, and Frailty Phenotype, operationalizing and feasibility may be challenging due to time constraints and the testing familiarity of the practitioner performing the assessment, as well as the patient’s participation [5]. Grip strength is one element of frailty, as the loss of grip strength has been correlated with increased disability, morbidity, and mortality, as well as increasing chronological age [6-8]. While grip strength has been used as a component of larger frailty scales, it is not known to what extent a simple grip strength assessment would compare with the more comprehensive frailty assessment tools. In this cross-sectional cohort study, we sought to identify the correlation between grip strength with validated frailty measures. Our null hypothesis was that there is no difference in the ability to detect frailty between grip strength and validated preoperative measures of frailty. In addition, we sought to identify whether grip strength correlates with age, the number of comorbidities, BMI, and the American Society of Anesthesiologists (ASA) Physical Status (ASA PS) class.

## Materials and methods

Study design and selection criteria

Following approval by the New York Medical College (NYMC) institutional review board, patients presenting to the Westchester Medical Center (WMC) Pre-Procedural Testing Clinic for surgery from June 1, 2021, to July 30, 2021, were given the opportunity to participate in the study (New York Medical College (NYMC)/WMC IRB (No.14462)). Exclusion criteria included any functional deficits (i.e. unilateral weakness post-cerebrovascular accident (CVA)) that prevented a participant from gripping a dynamometer repeatedly and reliably. In addition, participants with known muscular or neurological deficits to either the arm or hand were excluded from the study. Those who met the study criteria and provided informed consent were enrolled. The participant pool comprised consenting males (n = 39) and females (n = 34) ≥ 65 years of age who presented for elective surgery that did not involve the upper extremities. The study and all physical testing were carried out on weekdays: Monday-Friday from June 2021-July 2021 by one investigator (D.S.).

The investigator first evaluated the participant on the Clinical Frailty Scale (CFS-Investigator or CFS-I), which is a pictorial measure of frailty [9]. It consists of nine visual silhouettes of individuals of different levels of fitness; beginning from “1” for “Very Fit” to “5” for “Living with Mild Frailty” to “9” for “Terminally Ill” [9]. Next, the participant was asked about their interpretation of their own position on the spectrum of the Clinical Frailty Scale (CFS-Participant or CFS-P) [9]. The investigator recorded the participant’s response. Participants were asked to complete the FRAIL (Fatigue, Resistance, Aerobic capacity, Illnesses, and Loss of weight) score, a five-item questionnaire developed from the Fried Frailty Phenotype [10]. These items include questions about fatigue, resistance, ambulation, illness, and unintended weight loss. Participants scoring 3-5 points were labeled as frail, 1-2 points represented pre-frailty, and 0 represented robust health. This concluded the participant interview portion of the study. Lastly, participants in the study were asked to sit in a chair with their elbow positioned at 90 degrees and squeeze a digital dynamometer (Jamar Smart Digital Hand Dynamometer) three times with their dominant hand. The average of the three readings of grip strength was calculated and recorded. Frailty was defined as <30 kg of producible force for males and <20 kg of producible force for females, as defined in previous studies on frailty and sarcopenia [11,12]. If participants had one of their consecutive grip strength measurements vary by greater than 30%, the participant was asked to complete an additional round of grip strength measurement. The dynamometer tests were administered by a single investigator (D.S.)

Ethical considerations

The study was approved by NYMC/WMC IRB (No.14462). In order to participate in the study, participants who met inclusion criteria were approached, introduced to the study, and provided written informed consent.

Statistical analysis

Subjects were defined in a binary manner as “Frail'' by a grip strength measurement of <20 kg for females and <30 kg for males or “Non-Frail” if their grip strength measurement was ≥20 kg (females) and ≥30 kg (males). Frailty status as determined by CFS was defined as a CFS of 4 “Living with very mild frailty” or above and applied to both Clinical Frailty Scale as determined by Investigator (CFS-I) and Clinical Frailty Scale as determined by Participant (CFS-P). Lastly, participants scoring 3-5 points on the five-item FRAIL questionnaire score were determined to be frail. Male and female participants were analyzed separately due to the different measurement cut-offs for grip strength. All statistical analyses were performed using IBM SPSS Statistics version 28.0 (IBM Corp., Armonk, NY) and Microsoft Excel 2021 (Microsoft Corporation, Redmond, WA). Patient characteristics in the Frail and Non-Frail groups were compared using Mann-Whitney U-tests for categorical variables (ASA PS and comorbidities) and student t-tests for continuous variables (age, BMI, and grip strength). Spearman correlation coefficients were used to compare results from the CFS-I, CFS-P, and FRAIL scores in relation to grip strength and in relation to age. All p-values <0.01 were considered statistically significant. Cohen’s kappa was calculated between two categorical frailty measures using a 95% Confidence Interval (CI), in order to determine the percent agreement between measures. Cohen’s kappa was analyzed using Cohen’s kappa model of interpretation: weighted kappa value <0.20 = poor agreement, 0.20-0.40 = fair agreement, 0.41-0.60 = moderate agreement, 0.61-0.80 = substantial agreement, and 0.81-1.00 = almost perfect agreement [13]. Patient characteristics were presented in a descriptive table using Mann-Whitney U-tests and student t-tests, frailty measures were compared with grip strength between male and female participants using Spearman correlation coefficients, and Cohen’s kappa analysis was used to analyze the percent agreement between the frailty measures. Sensitivity, specificity, positive predictive value (PPV), negative predictive value (NPV), and likelihood ratio were calculated for grip strength versus the frailty scores for clinical application. In addition, a multivariate linear regression including both sexes was completed to test if BMI, age, ASA PS class, and the number of comorbidities correlated with grip strength. In addition, we categorized participants into two surgical risk groups depending on the type of surgery: low risk and intermediate/high risk, according to the American College of Cardiology (ACC)/American Heart Association (AHA) guidelines [14].

Internal pilot study

This study had an initial internal pilot study phase (n = 35) from which a power analysis was derived and determined the minimum sample size. During the pilot study, it was discovered that while females required n = 27 to reach statistical significance, males required an impractically high number to reach statistical significance (n = >40,000). Therefore, the projected sample size for males was kept at 27 as well to emphasize the difference between females and males in regard to correlations with frailty. The study was not designed to compare the strength of association of correlation coefficients between males and females. The only statistical comparisons between males and females related to the demographic measures of age, grip strength, and surgical risks. Therefore, the sample size was calculated based on the female counterpart. For the female calculations, the alpha (two-tailed) = 0.05, beta = 0.2, and expected correlation coefficient = 0.518. The most conservative correlation coefficient in females was used to calculate the necessary sample size. Analysis of Variance (ANOVA) (F-test for the difference in variance) was performed to determine if it would be possible to combine the pilot (n = 35) subjects with post-pilot study data. The ANOVA (F-test) revealed no significant differences in variances; therefore, it was determined acceptable to combine pilot data with post-pilot data to complete the data set.

## Results

Baseline characteristics

**Figure 1:**
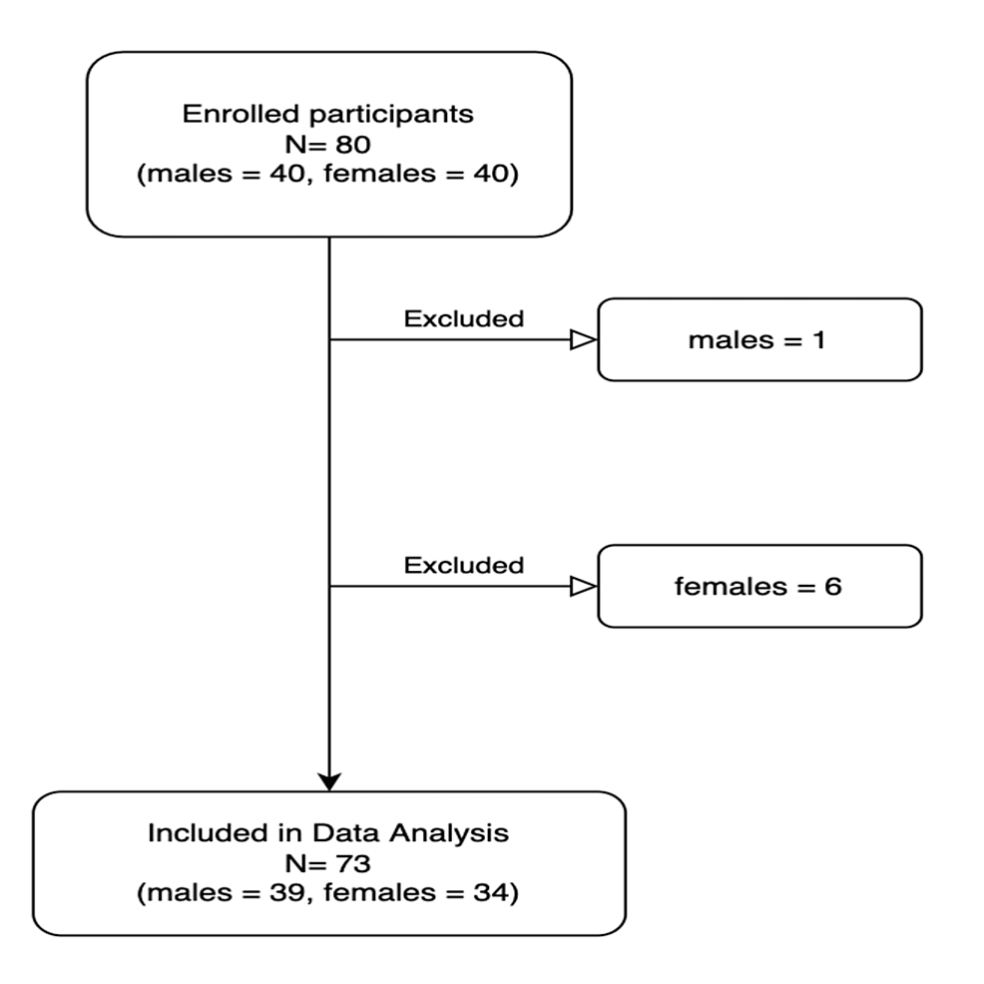
Flowchart of study inclusion and exclusion Flowchart of individuals included or excluded from the study. Excluded individuals either had their surgeries canceled or did not receive surgery by the end of the study period.

**Figure 2:**
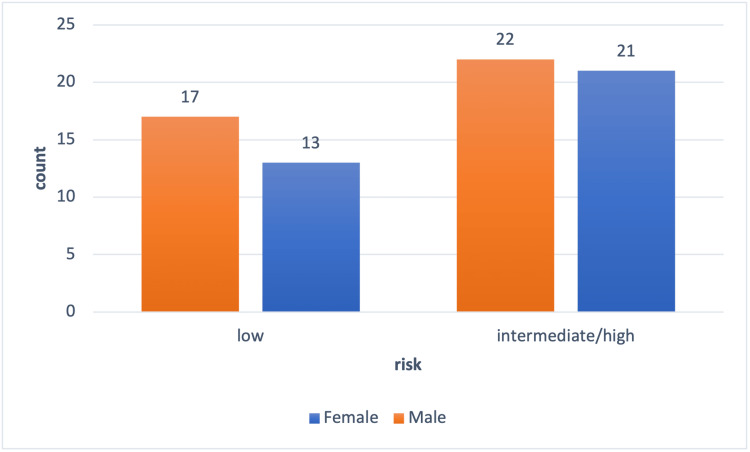
Surgery risk by gender The ratio of females and males who underwent risk-specific surgery; intermediate/high risk = males 22/39 (56%), females = 21/34 (62%); low risk = males 17/39 (44%), females = 13/34 (38%). Risk of surgical intervention as defined by the American College of Cardiology (ACC)/American Heart Association (AHA) guidelines.

The study had a final sample size of 73 participants out of 80 participants originally included (Figure 1). Seven individuals were excluded from the study due to surgery cancellations, incomplete chart data, or no surgery prior to the completion of the study. The final sample size of 73 was composed of 34 females (47% female) and 39 males (53% male). There was no significant difference in the average age of males and females (mean age = 74 years). Grip strength was significantly higher for males (mean = 32.2 kg, SD = 10.6 kg) than for females (mean = 20.6 kg, SD=5.6 kg, p = <0.0001). The chi-squared test for proportions found that there was no significant difference in surgery risk type between males and females (Figure 2).

Female characteristics

There was a significant negative correlation between female participants’ ratings on all three frailty scales versus grip strength as calculated by the Spearman correlation (Table 1). Our data represents an inverse relationship between female grip strength and frailty. As a result, higher female grip strengths correlated with less frailty or a lower frailty score; lower female grip strength correlated with more frailty or a higher frailty status. The significant negative correlation between female participants’ ratings on the CFS-I, CFS-P, and FRAIL scores versus participant grip strength is ranked in order of decreasing statistical significance (Table 1). A correlation was considered significant at the 0.01 level. It was found that the investigator’s CFS rating of the participant showed a stronger negative correlation than the participants’ CFS rating of themselves. CFS-I shared the strongest inverse relationship with grip strength, Spearman Rho = -0.776 (Table 1). As a result, an inverse relationship between female grip strength and frailty status was established; frail females were observed to have low grip strengths. Cohen’s kappa was performed to measure the percentage of agreement between the different frailty scales. We measured frailty status as decided by grip strength against frailty status as determined by the CFS-I, CFS-P, and FRAIL scores (Table 2). The agreement for female participants' CFS-I versus grip strength was rated as fair at the 0.40 Cohen's kappa level; the agreement for female participants' FRAIL versus grip strength was rated as moderate at the 0.54 Cohen’s kappa level (Table 2). In order to assess clinical applicability, sensitivity, specificity, PPV, NPV, positive likelihood ratio (PLR), and negative likelihood ratio (NLR) were calculated between grip strength and the CFS-I, CFS-P, and FRAIL scores. Specifically, CFS-I, CFS-P, and FRAIL scores were evaluated for their sensitivity, specificity, PPV, NPV, and likelihood ratios for predicting frailty as established by grip strength. The frailty scales also had their predictive and precision values calculated based on their agreement with grip strength. The study found that female CFS-I has the following: sensitivity 46.2%, specificity 90.5%, PLR 4.85, NLR 0.595, accuracy 73.5%, PPV 46.7%, and NPV 90.3% for predicting grip strength. As a result, if a participant is identified as not frail by CFS-I, there is a 90.5% chance that grip strength will also identify the participant as not frail. As the accuracy was 73.5%, there is a 73.5% chance that CFS-I will agree with frailty determined by grip strength. The study also found that female FRAIL score has the following: sensitivity 61.5%, specificity 90.5%, accuracy 79.4%, PLR 6.46, NLR 0.425, PPV 53.9%, and NPV 92.9%. As a result, if a participant is identified as frail by the FRAIL score, there is a 61.5% chance that grip strength will also identify the participant as frail. If a participant is identified as not frail by the FRAIL score, there is a 90.5% chance that grip strength will also identify the participant as not frail. The accuracy of the FRAIL score is 79.4%, meaning that there is a 79.4% chance that there will be agreement on frailty status between the FRAIL score and grip strength. In addition, the PLR is calculated based on the likelihood of having a disease (in this case, frailty) based on a positive test. The scale is 1-2 minimal likelihood, 2-5 small likelihood, 5-10 moderate likelihood, and >10 large likelihood. The PLR of the FRAIL score is >5; therefore, there is a moderate likelihood that frailty status by FRAIL score is predictive of frailty by grip strength. CFS-I has high specificity but has lower sensitivity than the FRAIL score. Out of the three measures of frailty (CFS-I, CFS-P, FRAIL score), the FRAIL score for females had the highest sensitivity. Overall, in female participants, CFS-I and FRAIL scores showed the greatest agreement with grip strength and the ability to rule out frailty (Table 3).

**Table 1:** Grip strength compared with assessed frailty scales Spearman’s correlation coefficient for males and females *denotes significance at 0.05, **denotes significance at 0.01 CFS-I = Clinical Frailty Score of Investigator, CFS-P = Clinical Frailty Score of Participant, FRAIL = Fatigue, Resistance, Aerobic capacity, Illnesses, and Loss of weight (FRAIL) score

	Rho	Sig. (2-tailed)
Female		
Grip strength vs CFS-I	-0.776**	6.82 •10^-8^
Grip strength vs CFS-P	-0.576**	3.69 •10^-4^
Grip strength vs FRAIL	-0.565**	4.96 •10^-4^
Male		
Grip strength vs CFS-I	-0.382*	0.016
Grip strength vs CFS-P	-0.276	0.085
Grip strength vs FRAIL	-0.129	0.430

**Table 2:** Agreement between grip strength and other frailty measures Summary of agreement between frailty measurements *denotes significance at p <0.05, ** denotes significance at p <0.01 CFS-I = Clinical Frailty Score of Investigator, CFS-P = Clinical Frailty Score of Participant, FRAIL = Fatigue, Resistance, Aerobic capacity, Illnesses, and Loss of weight (FRAIL) score

Frailty measurement	Cohen’s kappa (95% CI)	
	Male	Female
Grip strength vs CFS-I	0.08 (0.00-0.34)	0.40 (0.08-0.71) *
Grip strength vs CFS-P	0.14 (0.00-0.40)	0.28 (0.00-0.33)
Grip strength vs FRAIL	0.13 (0.00-0.24)	0.54 (0.25-0.84) **

**Table 3:** Predictive and precision values of frailty scales with grip strength frailty as the outcome Sensitivity (Sens), Specificity (Spec), Positive Likelihood Ratio (PLR), Negative Likelihood Ratio (NLR), Accuracy, Positive Predictive Value (PPV), Negative Predictive Value (NPV) for males and females comparing the agreement on frailty status among CFS-I, CFS-P, FRAIL score, and grip strength frailty. CFS-I = Clinical Frailty Score of Investigator, CFS-P = Clinical Frailty Score of Participant, FRAIL = Fatigue, Resistance, Aerobic capacity, Illnesses, and Loss of weight (FRAIL) score

	Sens (%)	Spec (%)	PLR	NLR	Accuracy (%)	PPV (%)	NPV (%)
Female							
CFS-I	46.2%	90.5%	4.85	0.595	73.5%	46.7%	90.3%
CFS-P	46.2%	81.0%	2.42	0.665	67.7%	30.4%	89.3%
FRAIL	61.5%	90.5%	6.46	0.425	79.4%	53.9%	92.9%
Male							
CFS-I	22.2%	85.7%	1.56	0.907	56.4%	21.9%	85.9%
CFS-P	27.8%	85.7%	1.94	0.843	60.0%	26.0%	86.8%
FRAIL	22.2%	90.5%	2.33	0.860	60.0%	26.0%	86.6%

Male characteristics

There was no significant correlation between male participants’ ratings on all three frailty scales versus grip strength (Table 1). The correlation was considered significant at the 0.01 level. In terms of Cohen's kappa, the agreement for male participants was rated as poor across all three frailty scales versus grip strength (Table 2). The sensitivity, specificity, PPV, NPV, and likelihood ratios as predicted by male CFS-I, CFS-P, and FRAIL scores were inferior when compared to corresponding scores for the female participants (Table 3). In particular, male participant frailty scales were weakest in sensitivity and PPV of CFS-I and FRAIL with values <45%, when compared to female participant scores (Table 3).

Combined characteristics

Multivariate linear regression was used to test if BMI, age, ASA PS class, and the number of comorbidities predicted grip strength in both males and females (Table 4). The overall regression was statistically significant, and it was found that age (p = <0.001) significantly correlated with grip strength (Table 4). Furthermore, it was found that BMI, ASA PS class, and the number of comorbidities did not significantly predict grip strength (p = >0.10). In addition, we examined the prevalence of frailty in females as determined by grip strength, CFS-I, CFS-P, and FRAIL score (Table 5). We found that, on average, frailty was more common in females than in males. However, grip strength rated more participants, both male (44%) and female (38%), as frail compared to any of the other frailty measures. The four-way Pearson’s chi-squared test for proportions revealed no significant difference between the proportions of frail versus not frail in the female cohort, as rated by the four different frailty scales (grip strength, CFS-I, CFS-P, and FRAIL score). Therefore, in the female cohort, grip strength rated frailty comparably to the other three frailty scores. The four-way Pearson’s chi-squared test revealed a significant difference in the proportions of male participants rated as frail versus not frail among the four different frailty scales (p = 0.013). This suggests that male grip strength frailty values differed significantly from the frailty scores given by the other three frailty scales (Table 5).

**Table 4:** Four-factor regression analysis for frailty status determined by grip strength For the four factor predictor model, R2=0.20, F(4,68) = 9.52, *p <0.001 B = Unstandardized Regression Coefficient, SE B = Standard Error of B, β = Standardized regression coefficient

Variable	B	SE B	β
Four-factor predictor model			
Constant	69.01	14.2	
Age	-0.65	0.17	-0.43*
BMI	0.09	0.23	0.04
ASA class	2.23	1.99	0.13
Comorbidities	-0.20	0.15	-0.15

**Table 5:** Ratio of frailty in males and females as identified by different frailty measurements The ratio and percentage of individuals identified as frail by grip strength, CFS-I, CFS-P, and FRAIL score. *Pearson chi-square for males revealed a significant difference (p = 0.013) in proportions of frailty between all four frailty scales: suggesting grip strength to be an outlier. There was no significant difference in the proportions of frailty between all four frailty scales for females. CFS-I = Clinical Frailty Score of Investigator, CFS-P = Clinical Frailty Score of Participant, FRAIL = Fatigue, Resistance, Aerobic capacity, Illnesses, and Loss of weight (FRAIL) score

	Grip strength	CFS-I	CFS-P	FRAIL
Female	13/34 (38%)	8/34 (24%)	10/34 (29%)	10/34 (29%)
Male	17/39 (44%)*	7/39 (18%)	8/39 (21%)	6/39 (15%)

## Discussion

Key findings

Despite the predictive insight that is afforded by the use of a pre-procedural frailty assessment, a frailty assessment has not become part of routine screening in most institutions [15]. The most commonly cited reasons for not implementing a pre-procedural frailty assessment have been (1) “lack of time” and (2) “lack of appropriate tools to measure frailty clinically” [16]. We, therefore, proposed to explore the utility of grip strength as a measure of frailty for individuals ≥65 years of age presenting for elective surgery. In this cross-sectional study, we determined that female participants, but not male participants, had grip strength measurements that significantly correlated with all of the tested frailty scales (CFS- I, CFS-P, and FRAIL). Previous research has found that females score higher on frailty indexes than males; females, however, live longer than males. This is known as the male-female health-survival paradox. There are many theories for this phenomenon, including the idea that adverse health conditions influence the risk of mortality in different ways for males versus females [17]. On one end, estrogen has been shown to have cardioprotective effects leading to lower cases of atherosclerosis and lipid profiles [18]. On the other end, estrogen has been associated with an increased risk of autoimmunity and chronic inflammation that may lead to more visible frailty phenotypes such as sarcopenia and cognitive impairment [18]. Post-menopause, estrogen levels decrease, which may lead to poor musculoskeletal health, which has been linked with not only increased muscle breakdown but also decreased contractile function [19]. One study found that in female muscle tissue, there was an abundance of estrogen receptors expressed specifically in type II muscle fibers [19]. Type II muscle fibers may also provide maximum hand contraction in situations such as during the measurement of grip strength. In addition, type II muscle fibers have been implicated in quick response muscle contractions in response to exogenous stressors [19]. Therefore, a decrease in type II muscle fiber responsiveness in women has been suggested as a reason for the increased incidence of falls in females compared to males [19]. Furthermore, a study by Leyk et al. found that female athletes, despite being highly trained in handgrip active sports, matched only the 25th percentile of athletically untrained men [20]. This study calls into question the potential differences in muscle composition between males and females. A recent study by Canales et al. proposed the use of point-of-care ultrasound (POCUS) as a novel tool for the identification of frailty [21]. In their study, quadricep dimensions were visualized using POCUS and subsequently related to a participant’s BMI to reveal the true muscle ratio. The utilization of ultrasound on upper body muscles is representative of the difference in the distribution of muscle mass between males and females.

Another consideration is the existence of different domains of frailty: physical, psychological, cognitive, and social [22]. Our study focused on the physical domain of frailty through the measurement of grip strength; the lack of significance between male grip strength and frailty only accounts for physical frailty and leaves the other domains to be explored. In addition to biological factors, social factors, such as the frequency of healthcare utilization, should be analyzed. Females are more likely than males to access healthcare, and this may play a role in preventative health as well as an early intervention [18]. As a result, females may not only display frailty status more readily, but they are also more likely to utilize healthcare and benefit from early intervention and prevention. It is also possible that grip strength is a surrogate for post-menopausal estrogen losses, which may also be indicative of frailty.

To further evaluate the finding that male grip strength was not significantly correlated with the frailty scales, we decided to correct for BMI. Previous literature indicates a sliding cut-off for grip strength in males, due to differences in BMI, and even offers different optimal cut-off points for grip strength based on BMI [23]. Other more recent studies suggest measuring grip strength in the participant’s nondominant hand and having substantially lower cut-offs for grip strength [21]. For instance, in 2022, Canales et al. reported that the cut-off of frailty for males (20.5 kg - 23 kg) and females (11.5 kg - 13 kg) for nondominant hand grip strength based on BMI quartiles was substantially lower than dominant hand cut-offs of <20 kg for females and <30 kg for males [21]. Despite statistical corrections for BMI and age in males, we did not obtain a statistically significant correlation between male dominant grip strength trends and the frailty scales. Grip strength is not as useful a tool for identifying frailty in men as it is in women. In female participants, as their frailty status increased, their recorded grip strength decreased. However, no significant relationship or correlation was observed in males between their grip strength and frailty measurements. We suspect this is due to the genetic, hormonal, muscular, and environmental differences between males and females, which were not accounted for in this study and require further exploration.

Our study identified age as the only variable that significantly predicted grip strength; BMI, ASA PS class, and the number of comorbidities did not significantly predict grip strength for males or females. With regards to the ASA PS class, literature has called into question the inter-rater reliability between anesthesiologists for assigning an ASA PS class; this may also impact its correlation with grip strength and frailty [24]. In 2014, Riley et al. surveyed 151 anesthesiologists on 10 hypothetical patient profiles and concluded that, for ASA PS class, the inter-rater agreement according to Cohen’s kappa was 0.40, which depicted fair agreement at best [24]. This study further found that none of the patient profiles shared complete unanimity, and only one patient profile received a variation that was limited to only two ASA PS class scores [24]. A previous study surveyed 97 anesthesiologists in a similar fashion on 10 hypothetical patient profiles and found the inter-rater agreement varied between Cohen’s kappa 0.21 - 0.40 for overall fair agreement [25].

When evaluating CFS, there were two components considered: CFS-I and CFS-P. Having both CFS-I and CFS-P allowed for a comparison to be made between patient/self-identified CFS and investigator-assessed CFS. Self-rated health, defined as a patient assessment of their health status, has been used as a reliable predictor of mortality [26]. When a frailty index was constructed from a self-reported health deficits questionnaire, it was found to be associated with adverse health outcomes [27]. Acknowledging the emerging value of self-rated health, we compared whether self-rated health or investigator-rated health would more significantly correlate with grip strength. Our study found that grip strength correlates more significantly with frailty when the frailty status is assessed by the investigator instead of the participant. This may reflect the importance of provider participation in the frailty assessment when using grip strength in the preoperative population.

Study limitations

A general limitation of our study is that there is no established gold standard for frailty measurements to use as a reference [28]. We chose CFS, FRAIL score, and grip strength as measures of frailty due to their previously proven validity, reliability, ease of use, and minimum test administration time requirement. Another limitation of our study is that, because this is a cross-sectional cohort study, frailty measurements are single points in time. In addition, relating the less commonly studied variable of CFS-P to future outcomes would help elucidate its potential as a component of CFS.

Certain study variables, such as comorbidities and ASA PS class, were obtained from electronic medical records and were not determined by the investigator. The number of comorbidities was particularly susceptible to clinician bias because only the number of comorbidities, and not the severity of each of the comorbidities, was used in the study. In addition, we investigated a limited number of study variables; other potential risk factors for frailty, such as socio-economic status, nutrition status, and mental wellness, were not evaluated [29]. In regards to nutrition, a nutritional status below the requirements for sustainment could lead to sarcopenia and eventually frailty. Another limitation of this study is that the study was limited to ambulatory or ambulation-aided patients. Participants who were terminally ill or bed-bound were therefore excluded from the study. The use of predefined cut-offs for grip strength rather than monitoring the decline of grip strength is a limitation of the cross-sectional nature of the study. Predefined cut-off values for grip strength may not accurately portray an individual’s position on the frailty spectrum, as would observing a decline in their baseline grip strength. In our study, we observed that grip strength was also the only frailty measure in which there was a higher percentage of frailty in males versus females. We believe this finding may be due to the subjectivity of grip strength cut-off values or the lesser importance of dominant hand grip strength. Another limitation of this study is the lack of a clinical outcome. Our study focuses on exploring the concordance between the frailty measures. We are limited to comparing frailty scores against one another and against grip strength, as opposed to a positive or negative clinical outcome as a result of frailty identified by different measures.

## Conclusions

This single-center, cross-sectional cohort study examined grip strength as a measure of frailty in elderly patients. Our findings demonstrate that hand grip strength is significantly correlated with the CFS-I, CFS-P, and FRAIL scores for females but not for males. For both genders, age is a significant independent predictor of grip strength. BMI, ASA PS class, and the number of comorbidities did not significantly predict grip strength. Ultimately, our results indicate that grip strength may provide utility in preoperative screening for frailty in females only. Further studies are needed to clarify the lack of correlation between grip strength and frailty in males as well as the value of the non-dominant hand grip strength as a measure of frailty. Additionally, the correlation of preoperative grip strength with post-surgical outcomes should be studied and may further validate the preoperative utility of grip strength, particularly in female patients.
